# Effectiveness of pre-operative chemical component separation with computed tomography-guided intramuscular injection of OnabotulinumtoxinA in outcomes of large complex incisional ventral abdominal hernia repair: a propensity score-weighted comparative analysis

**DOI:** 10.1007/s10029-025-03369-w

**Published:** 2025-05-23

**Authors:** Younes Jahangiri, Dylan Goldsmith, Amy Banks-Venegoni, Gregory Fritz, Giuseppe Zambito, Albert Jiao, Lane King, Jordan Castle, Khaleel Quasem, James Morrison

**Affiliations:** 1 Division of Interventional Radiology, Corewell Health West Michigan, 100 Michigan St. NE, Grand Rapids, MI 49503 USA; 2 Department of Surgery, Corewell Health West Michigan, Grand Rapids, MI USA; 3 Department of Radiology, Brigham and Women’s Hospital, Harvard Medical School, Boston, MA USA; 4 Department of Anesthesia & Critical Care, The University of Chicago, Chicago, IL USA; 5 Division of Interventional Radiology, Inland Imaging, Spokane, WA USA; 6 College of Human Medicine, Michigan State University, Grand Rapids, MI USA; 7 Department of Radiology, Michigan State University, East Lansing, MI USA

**Keywords:** Complex hernia, Ventral hernia, Component separation, Botox, OnabotulinumtoxinA

## Abstract

**Purpose:**

To evaluate the effectiveness of chemical component separation (CCS) via computed tomography (CT)-guided intramuscular injection of OnabotulinumtoxinA (Botox) in postoperative recurrence of large complex incisional ventral abdominal hernias.

**Materials and methods:**

A total of 97 patients with large ventral abdominal hernias who underwent complex hernia repair between November 2017 and October 2021 after (*n* = 37) (Botox) or without (*n* = 60) Botox injection (no-Botox) were included in the study. Data were summarized as median [min–max] or frequency (%) and analyzed using the Fisher's exact test, Mann–Whitney U test, multivariate logistic regression with backward stepwise selection of covariates and augmented inverse probability-weighted analysis with Stata BE 18.0 at a significance level set at 0.10.

**Results:**

There was no statistically significant difference between Botox and no-Botox groups in patients’ age (64[34–78] vs. 62[24–94], *p* = 0.885), sex (females: 46% vs. 55%, *p* = 0.410), body mass index (BMI) (32[19–53] vs. 31[18–50], *p* = 0.431) and hernia volume (3197[226–24232] vs. 2366[140–24314], *p* = 0.458). Median follow-up duration was 38[2–72] months in Botox and 48[6–81] months in no-Botox groups (*p* = 0.010), and all-time hernia recurrence was 8% in Botox and 22% in no-Botox groups, respectively (*p* = 0.097). In multivariate regression analysis, CCS, hernia volume, implanted mesh type and overall postoperative complications were associated with hernia recurrence. After propensity score weighting for follow-up duration, surgical component separation and postoperative discharge destination, CCS was associated with 71% reduced risk of hernia recurrence (*p* = 0.045).

**Conclusion:**

The results of this study suggests that CT-guided chemical component separation with intramuscular injection of OnabotulinumtoxinA may be effective in reducing the risk of post-surgical recurrence of large complex incisional ventral abdominal hernias.

## Introduction

Large ventral hernias are defined as abdominal wall hernias with diameter ≥ 10 cm by the European Hernia Society [1, 2]. Recurrence is an important complication in the surgical repair of large, complex ventral hernias. The rate of incisional and ventral hernia repairs that recurred and required reoperation was substantial: up to 16.0% for the open surgical approach and 18.8% for the minimally invasive approach [3]. Factors that are predictive of hernia recurrence include smoking, diabetes, chronic obstructive pulmonary disease (COPD), ASA grade III–IV, steroid use, and the repair of incisional or recurrent hernias as opposed to primary hernias [4]. Intraoperative factors impacting recurrence rates include achieving fascial closure over bridged repair, using ventral mesh over sutures only, opting for synthetic over biologic mesh, and choosing laparoscopic over open repair [4].

Current management of this condition shows a wide variety of approaches. Despite most surgeons adhering to the Ventral Hernia Working Group's recommendations on preoperative optimization and lifestyle changes, such as smoking cessation more than four weeks before surgery, maintaining HbA1c below 7% in diabetic patients, and weight-loss regimens to achieve a BMI below 30 kg/m^2^, many surgeons were not deterred from operating if patients did not meet these targets [5]. Surgeons have adopted several practices to aid in achieving primary closure and reduce the risk of complex ventral hernia recurrence, including, surgical mesh placement, aponeuroplasty, pneumoperitoneum, tissue expanders, and surgical component separation – a release of the abdominal musculature to increase laxity [6].

Use of chemical component separations (CCS) with OnabotulinumtoxinA is a relatively recent advancement in the management of complex large ventral hernias, aiding the surgeon in achieving primary closure [7]. OnabotulinumtoxinA is a potent neurolytic toxin extracted from Clostridium botulinum, and is famous for well-known food-borne intoxication [8, 9]. This drug exerts its effect by induction of temporary flaccid paralysis of the abdominal wall musculature by presynaptic acetylcholine blockade, reaching its maximal effect by two weeks [10]. This surgical adjunct has been demonstrated to be safe and effective in creating abdominal wall laxity to decrease hernia width and assist in primary closure [10–12]. However, the current evidence directly associating this preoperative treatment with lower recurrence rates is sparse. The purpose of this study is to assess the efficacy of chemical component separation with OnabotulinumtoxinA in reducing the risk of recurrence of complex large ventral abdominal hernias after surgical repair.

## Materials and methods

### Patients

**Fig. 1:**
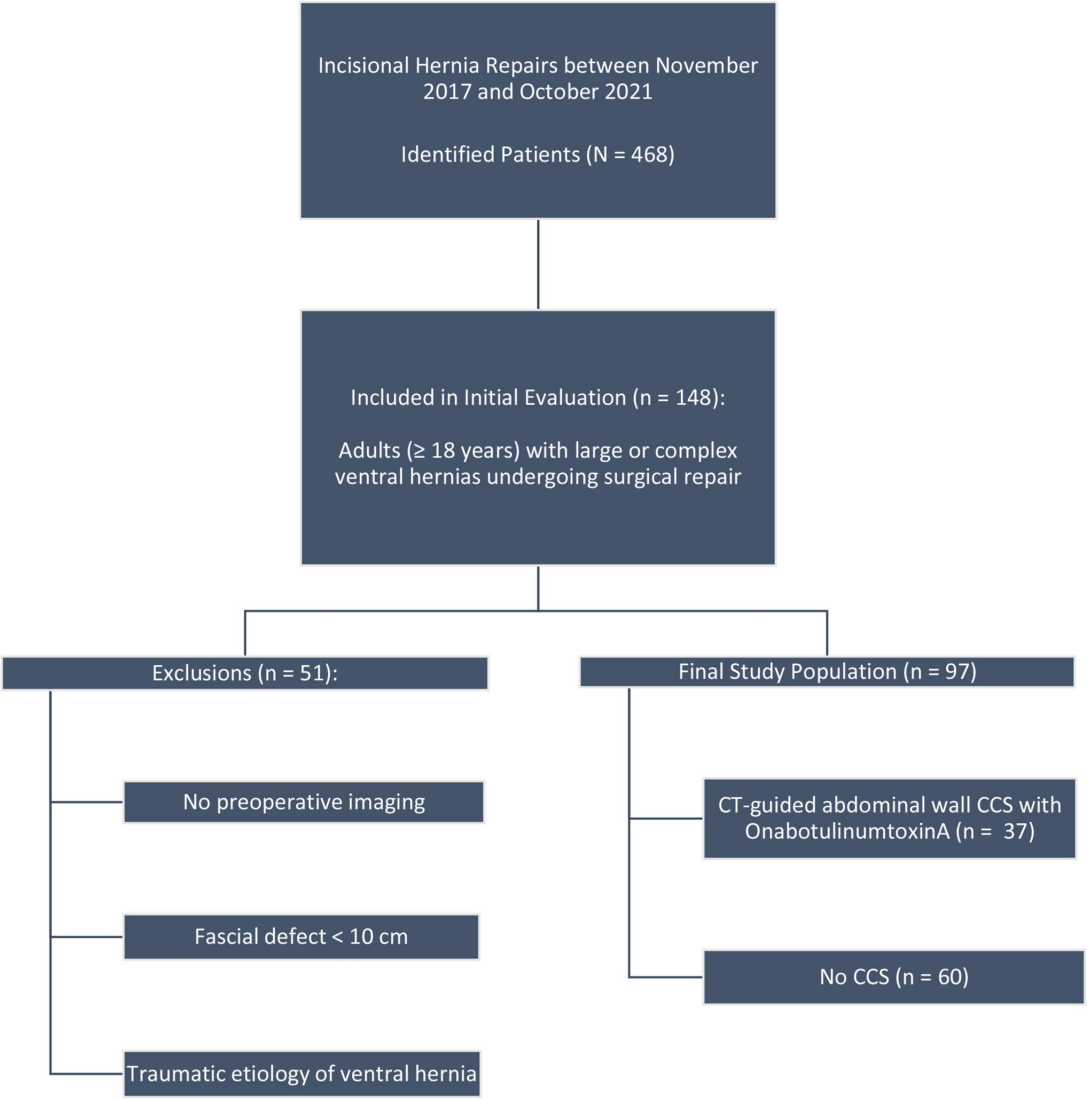
Flow Chart demonstrating study patient selection

This single-institution retrospective case control study​ was evaluated by the local Institutional Review Board (IRB) and a consent waiver was granted. The Local Electronic Medical Record system was queried using the CPT codes associated with incisional hernia repair (49560, 49561, 49565, 49566, 49585 and 49587). A total of 468 patients were identified who had incisional hernia repair between November 2017 and October 2021. All patients ≥ 18 years of age who had undergone surgical repair for large or complex ventral hernias were included in the initial evaluation (*n* = 148). After exclusion of cases without preoperative imaging, those with fascial defects measuring less than 10 cm or traumatic etiology of the ventral hernia (*n* = 51), 97 subjects were enrolled in the study. Among the enrolled subjects, 37 patients underwent preoperative CT-guided abdominal wall CCS with OnabotulinumtoxinA (Botox group) and 60 were those who had not received CCS (no Botox group) (Fig. 1). Patients presenting with fascial defects greater than 8 cm were considered candidates for chemical component separation using Botox injection. The decision to proceed with Botox injection was based on hernia size, a shared decision-making discussion between the surgeon and the patient, and the patient’s preference. Data regarding patients’ demographics, clinical status, clinical and imaging hernia characteristics, surgical technique, and postoperative follow-up were collected.

### Botox injection technique

**Fig. 2:**
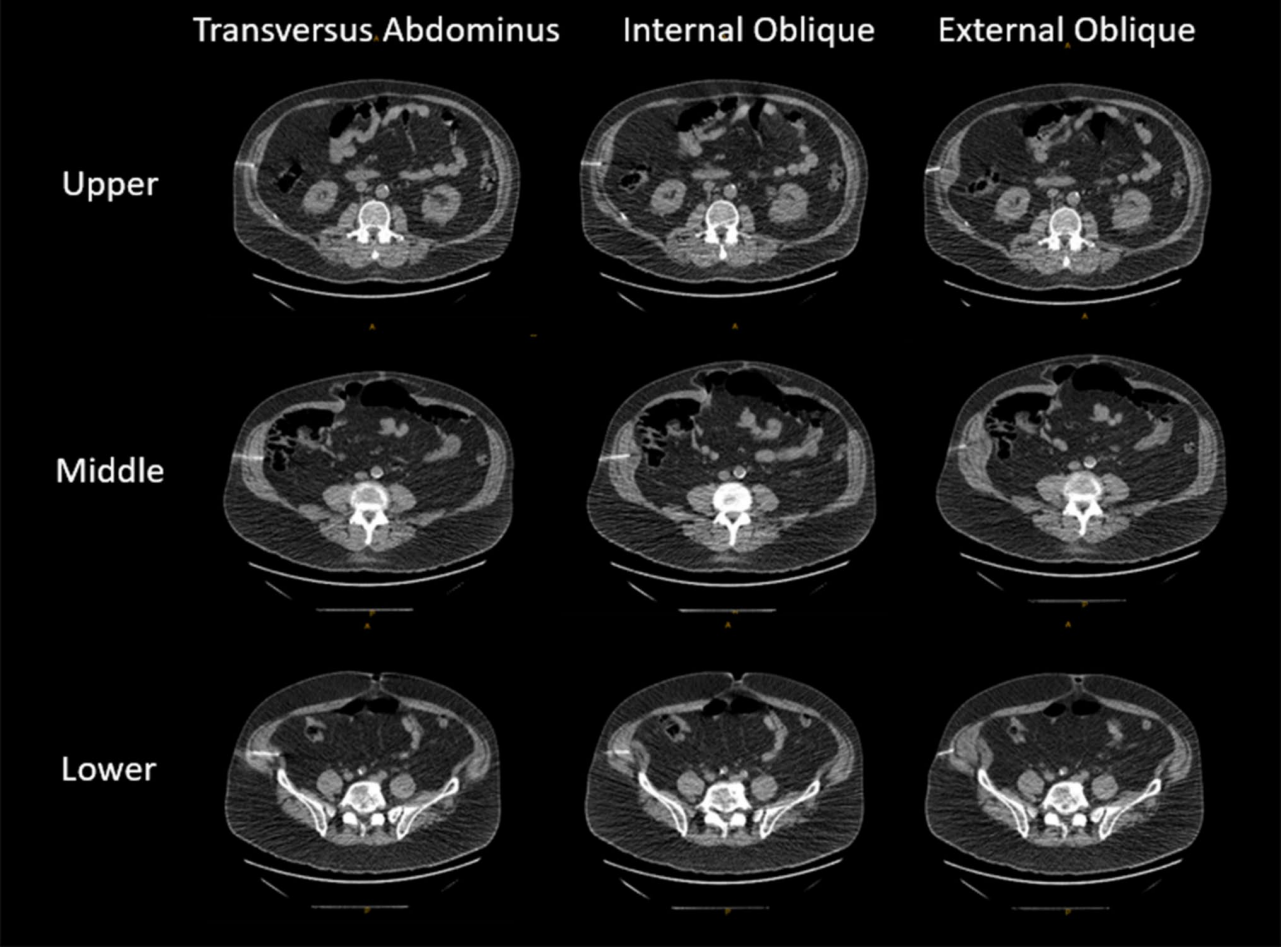
Computed tomography (CT)-guided injection of OnabotulinumtoxinA into the abdominal wall muscles, with three injection points (upper, mid, and lower) per muscle, totaling nine injections per side. The injections on the right side are shown here

A computed tomography (CT)-guided percutaneous bilateral injection of Botox into the abdominal wall musculature was performed for each patient in the Botox group using the technique described by Zielinski and Zendehas [13, 14]. Briefly, 300 international units of OnabotulinumtoxinA were diluted in 180 ml of sterile normal saline. Six total sites were selected—three on each side along the mid-axillary line: one immediately below the rib cage, one immediately above the iliac crest, and a third midpoint site equidistant between them. The sites were prepped using maximal sterile technique. Moderate sedation was provided with 1–2 mg midazolam and 50 μgr fentanyl with continuous monitoring. Local anesthesia was applied at each site. Under CT fluoroscopic guidance, a 22-guage needle was advanced into each of the external oblique, internal oblique, and transverse abdominis muscles at each site (superior, mid, and inferior bilaterally) (Fig. 2). After confirming negative test aspiration, 10 mL of diluted Botox serotype A was injected into each muscle at each site for a total of 18 injections. Post-injection imaging demonstrated fluid expansion within the muscle layers, confirming appropriate intramuscular delivery.

### Surgical repair technique

Under general anesthesia and sterile technique, a midline incision was created and the previous scar tissue was excised. The subcutaneous tissues were divided down to the fascia. Adhesions were lysed sharply and/or bluntly as needed to free any bowel or omentum from the abdominal wall and hernia sac. Any nonviable tissue was excised, and the hernia sacs were resected circumferentially to expose intact fascial edges. The specific surgical technique varied based on defect size, location, and surgeon preference. In cases where primary fascial closure was feasible without undue tension, a standard retrorectus (Rives-Stoppa) repair was performed. In cases with larger defects requiring additional myofascial advancement, posterior component separation via transversus abdominis release (TAR) was performed. External oblique release (anterior component separation) was used selectively when additional mobilization was required. Depending on the defect size and location, a suitable prosthetic or biologic mesh was selected and positioned in either the retrorectus (sublay) or underlay plane. Mesh fixation was achieved with a combination of transfascial sutures or running sutures, ensuring wide overlap of the defect. Posterior and anterior fascial layers were then closed, typically with running or interrupted absorbable (e.g., polydiaxanone (PDS)) sutures.

Incisional negative-pressure wound therapy was applied postoperatively per surgeon preference.

### Definition of terms

**Fig. 3:**
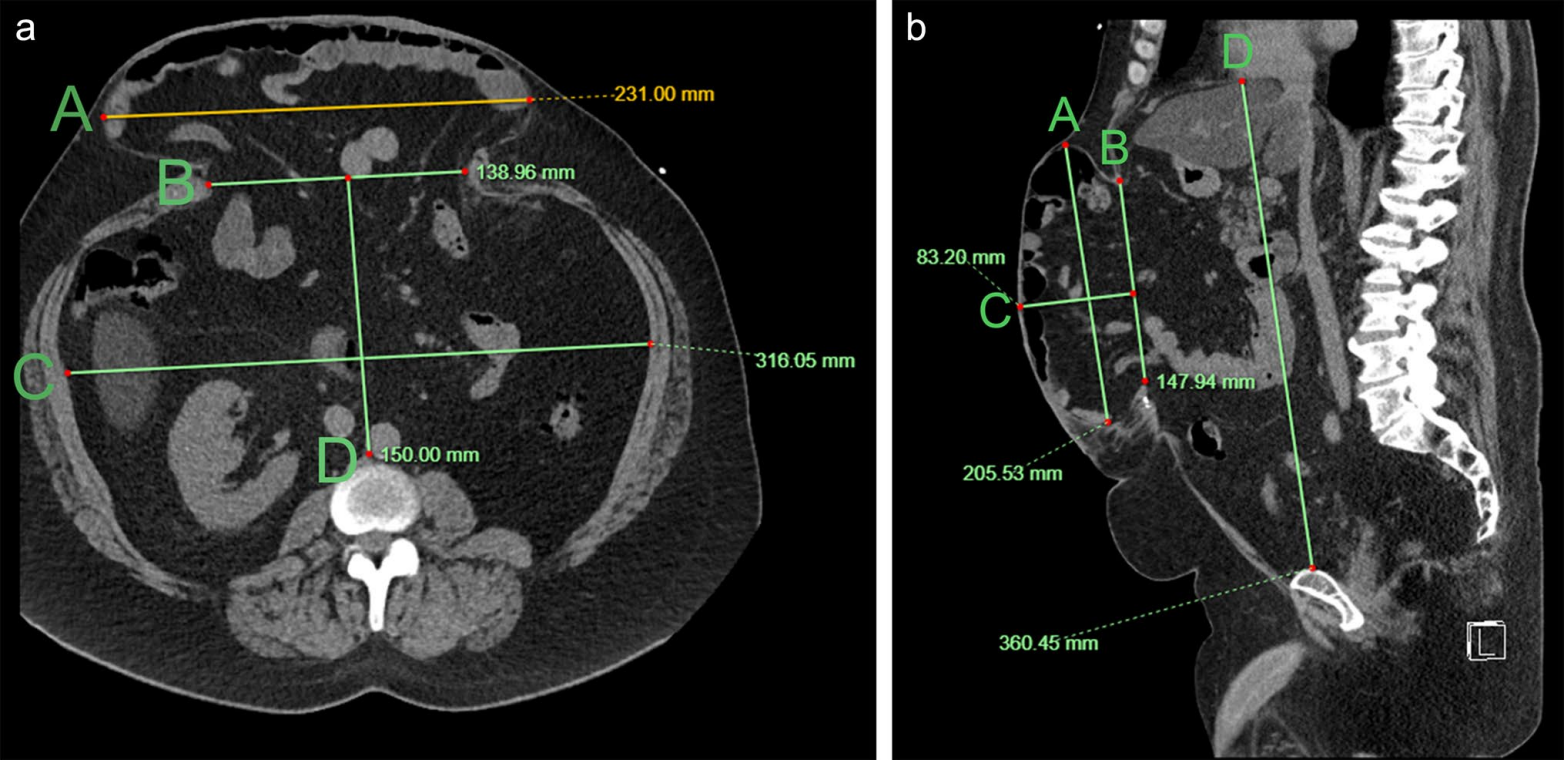
Non-contrast-enhanced abdominal computed tomography denoting measurements of abdominal and hernia cavities dimensions in axial (a) and sagittal (b) projections. On the axial projection (**a**), line A denotes the widest axial diameter of the hernia sac, line B is measured at the hernia neck, and lines C and D denote the widest transverse and anteroposterior abdominal cavity dimensions, respectively. On the sagittal projection (**b**), line A denotes the widest craniocaudal dimension of the hernia sac, line B denotes the widest craniocaudal dimension of the hernia neck, line C denotes the widest anteroposterior dimension of the hernia sac, and line D is measuring the widest craniocaudal dimension of the abdominal cavity from the diaphragm to the superior aspect of the pubis

Hernia and abdominal cavity dimensions were measured on the pre-surgical imaging (CT or MRI) according to the method suggested by Tanaka et al. [15] (Fig. 3). Hernia and abdominal cavity volumes were calculated using the formula for ellipsoid shapes, also suggested by the same group: $Volume=\frac{4}{3}\times \pi \times (length \times width\times height)$. All measurements were performed by a trained interventional radiology resident (DG).

Hernia location was classified as midline or lateral according to the European Hernia Society (EHS) 2009 classification [1]. Follow-up duration was defined based on any imaging or clinical follow-up documenting the status of treated hernia. All-time hernia recurrence was defined as documented recurrence of the repaired hernia during the entire postsurgical follow-up period. Clinical evaluation was performed during follow-up visits to assess for any signs or symptoms suggestive of hernia recurrence, such as bulging, pain, or functional impairment. Definitive hernia recurrence was objectively assessed using cross-sectional imaging (CT or MRI) to evaluate the integrity of the abdominal wall at the surgical site. Recurrence was defined as the presence of a full-thickness fascial defect with recurrent protrusion of the intra-abdominal contents, as confirmed on imaging.

### Statistics

Categorical and numerical data were summarized as frequency (percentage) or median (min – max), and were compared between two groups using the Fisher’s exact test or the Mann–Whitney U test, respectively.

To assess multivariate-adjusted association between chemical component separation using Botox injection with hernia recurrence, a multivariate logistic regression model was applied. A stepwise technique with forward variable selection approach was used. Given the significant difference in follow-up duration between treatment arms, the natural logarithm of follow-up time was fixed in the model as the offset variable. The entry and removal significance thresholds were set at 0.1 and 0.2, respectively. Between-covariate interactions were evaluated using the Pearson’s correlation coefficients with Bonferroni adjustment, and any statistically significant interaction terms were included in the final multivariate model.

Next, to minimize selection bias, a propensity score-weighted approach was conducted. For this purpose, an augmented inverse propensity-weighted (AIPW) model was constructed. This method is a doubly-robust technique that has been shown to carry less bias compared to other techniques of propensity score estimation such as regression or inverse probability weighting methods [16]. Potential confounders were selected based on their significant association with treatment (Botox) and outcome (hernia recurrence) as well as scientific inference. Any variable with standardized mean differences ≥ 0.20, were also included in the main model for a multivariate-adjusted model. Absolute and relative average treatment effects (ATE) of chemical component separation were quantified. Study power was calculated based on differences of hernia recurrence between Botox and no Botox groups. All analyses were performed with Stata BE 18.0, and the significance level was set at 0.10.

## Results

### Comparison between Botox and no Botox groups

The median time between Botox injection and surgical repair was 30 days (range: 8–58 days), and the median time between the pre-surgical imaging and hernia repair was 3.1 months (range: 0–26.9). A comparison of the treatment arms is presented in Table 1. There was no statistically significant difference between Botox and no Botox groups regarding patients’ demographics, pre-surgical clinical status and comorbidities. A higher number of patients in Botox group underwent surgical component separation (SCS) and also had home without need for assistance as their discharge destination compared to no Botox group (49% versus 22%, *p* = 0.007; and 92% versus 73%, *p* = 0.041, respectively). Primary fascial closure was achieved in 95% of the Botox group versus 92% of the non-Botox group (*p* = 0.705). The Botox group had a shorter total median follow-up duration (38 versus 48 months, *p* = 0.010) and lower hernia recurrence rates (8% versus 22%, *p* = 0.097). Analysis power at the significance level of 0.1 was 0.473.

**Table 1:** Comparison between subjects in Botox and non-Botox groups

	Pre-operative Botox injection^1^		
	No	Yes	*p*-value^2,3^
	*n* = 60 (62%)	*n* = 37 (38%)	
Age (years)	62 (24–94)	64 (34–78)	0.885
Sex			
Female	33 (55%)	17 (46%)	0.410
Male	27 (45%)	20 (54%)	
BMI (kg/m^2^)	31 (18–50)	32 (19–53)	0.431
Diabetes			
No	38 (63%)	27 (73%)	0.379
Yes	22 (37%)	10 (27%)	
CAD			
No	56 (95%)	32 (86%)	0.254
Yes	3 (5%)	5 (14%)	
Previous hernia repair			
No	36 (62%)	17 (46%)	0.142
Yes	22 (38%)	20 (54%)	
Previous mesh implantation			
No	35 (61%)	17 (55%)	0.651
Yes	22 (39%)	14 (45%)	
Smoking status			
Never	29 (48%)	16 (43%)	0.679
Current or former	31 (52%)	21 (57%)	
Recent smoking status			
Never smoked	45 (75%)	32 (86%)	0.205
Current smoker or quit during the past year	15 (25%)	5 (14%)	
Location of hernia			
midline	59 (98%)	36 (97%)	1.000
lateral	1 (2%)	1 (3%)	
Hernia Volume (cm^3^)	2366 (140–24314)	3197 (226–24,232)	0.458
Abdominal Cavity Volume (cm^3^)	50966 (19698–113636)	39157 (18982–112922)	0.238
Hernia-to-abdominal Cavity Ratio (%)	5 (0–48)	6 (0–99)	0.375
Complication associated with Botox injection			
No	na	37 (100%)	na
Botox-to-surgery time (days)	na	30 (8–58)	na
Retrorectus surgical technique			
No	15 (25%)	7 (19%)	0.620
Yes	45 (75%)	30 (81%)	
Primary fascial closure			
No	5 (8%)	2 (5%)	0.705
Yes	55 (92%)	35 (95%)	
Delayed closure			
No	53 (88%)	36 (97%)	0.150
Yes	7 (12%)	1 (3%)	
Surgical component separation			
No	**47 (78%)**	**19 (51%)**	**0.007**
Yes	**13 (22%)**	**18 (49%)**	
Application of mesh			
No	5 (8%)	3 (8%)	1.000
Yes	55 (92%)	34 (92%)	
Mesh type			
None	5 (8%)	3 (8%)	0.593
Polypropylene (Prolene)	24 (40%)	21 (57%)	
Synthetic Biodegradable (Vicryl, Phasix)	9 (15%)	3 (8%)	
Composite Mesh (Bard Ventralex)	14 (23%)	7 (19%)	
Biologic (Strattice)	8 (13%)	3 (8%)	
Additional intraoperative procedures other than hernia repair			
No	44 (73%)	30 (81%)	0.466
Yes	16 (27%)	7 (19%)	
Need for blood transfusion during surgery			
No	57 (97%)	37 (100%)	0.521
Yes	2 (3%)	0 (0%)	
Estimated Blood Loss (ml)	75 (15–1000)	100 (10–300)	0.434
Hospital stay (days)	3 (0–20)	4 (1–20)	0.688
Discharge destination			
Home	**44 (73%)**	**34 (92%)**	**0.041**
Home w/Assistance	**12 (20%)**	**1 (3%)**	
Subacute Rehab	**3 (5%)**	**1 (3%)**	
Inpatient rehab	**1 (2%)**	**1 (3%)**	
Any complications			
No	41 (69%)	24 (67%)	0.822
Yes	18 (31%)	12 (33%)	
Post-operative pain control issues			
No	57 (95%)	36 (97%)	1.000
Yes	3 (5%)	1 (3%)	
Reoperation for hernia repair during hospitalization			
No	60 (100%)	37 (100%)	na
30-day hernia reoperation			
No	60 (100%)	37 (100%)	na
Hernia recurrence during entire postop follow-up			
No	**47 (78%)**	**34 (92%)**	**0.097**
Yes	**13 (22%)**	**3 (8%)**	
Alive at last follow-up?			
Yes	60 (100%)	37 (100%)	na
Follow-up duration (months)	**48 (6–81)**	**38 (2–72)**	**0.010**

1. Data summarized as frequency (%) for categorical variables and median (min – max) for numerical values

2. Percentages are compared using the Fisher's exact test, and the median values are compared with the Mann–Whitney U test

3. Statistically significant differences are shown in bold. Statistical significance level is set at 0.10

### Subpopulation comparison (Botox-only versus surgical component separation only)

**Fig. 4:**
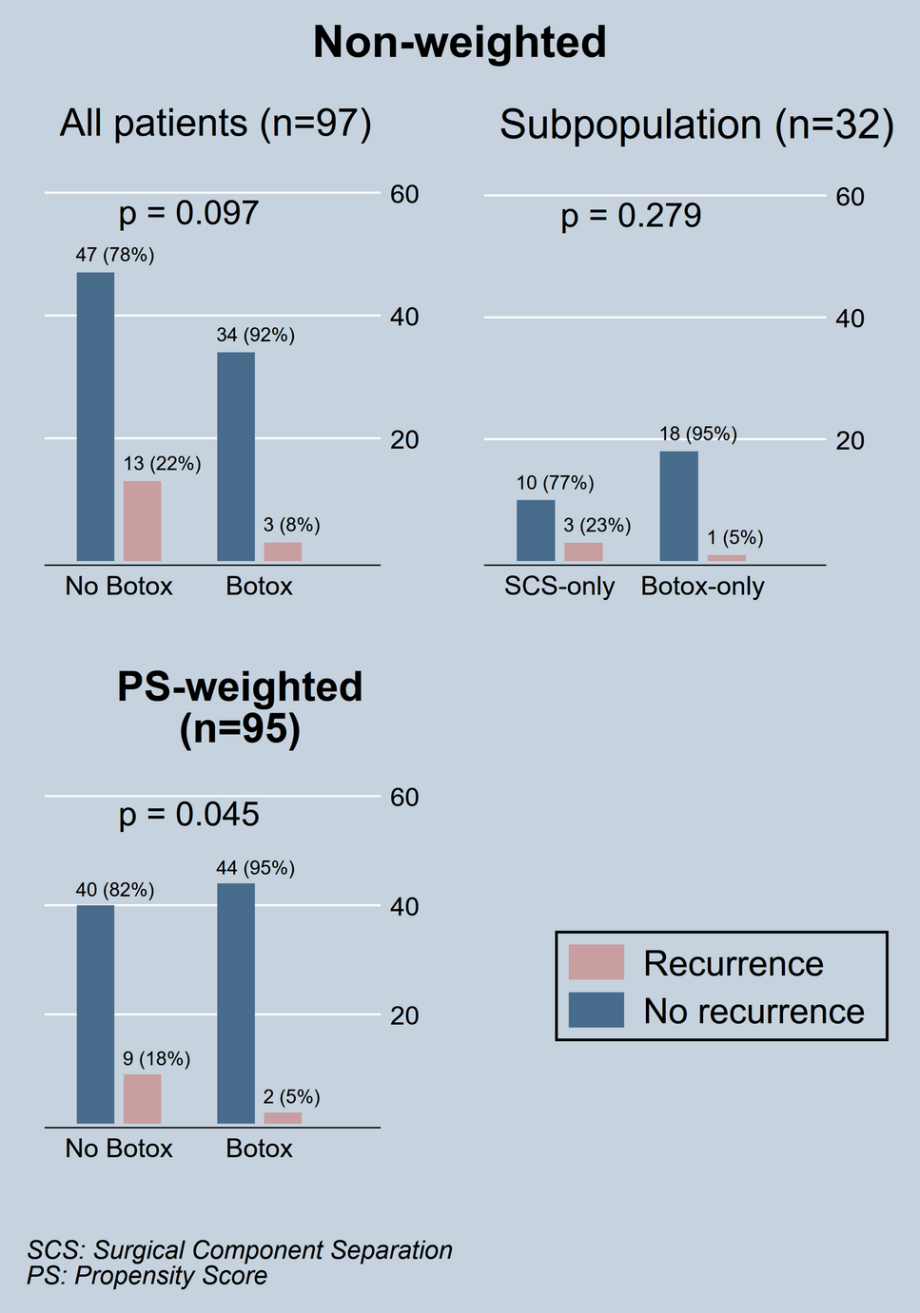
Comparisons of ventral hernia recurrence rates after surgical repair of large incisional ventral abdominal hernias. Both non-weighted and propensity score-weighted comparisons are presented. The figure highlights non-weighted comparisons for all subjects as well as a subgroup of patients treated with surgical component separation only, versus those treated with Botox (chemical component separation) without surgical component separation. Differences were assessed using the Fisher’s exact test, and *p* values are provided

There was no statistically significant difference in primary fascial closure rates neither between SCS and no SCS groups (94% versus 91%, *p* = 1.000) nor when Botox-only group was compared with the SCS-only group (91% versus 92%, *p* = 1.000). One out of 19 (5%) patients with Botox only treatment and 3/13 (23%) patients with surgical component separation only treatment had hernia recurrence on follow-up (*p* = 0.279) (Fig. 4). Analysis power for this comparison was 0.200 at significance level of 0.1.

### Factors associated with hernia recurrence

Patients with hernia recurrence during follow-up (16/97 (16%)) had larger median hernia volumes (4221 versus 2316 cm^3^, *p* = 0.077), higher rate of biologic mesh implantation (31% versus 7%, *p* = 0.020), longer median hospital stay (7 versus 3 days, p = 0.002), higher rate of home with assist as discharge destination (31% versus 10%, *p* = 0.088), and higher rates of postoperative complications (94% versus 19%, *p* < 0.001) including postoperative pain control issues (19% versus 1%, *p* = 0.014) (Table 2).

**Table 2:** Comparison between subjects with and without all-time hernia recurrence

	Hernia recurrence during entire postop follow-up^1^		
	No	Yes	*p*-value^2,3^
	*n* = 81 (84%)	*n* = 16 (16%)	
Age (years)	64 (24–94)	62 (33–78)	0.509
Sex			
Female	41 (51%)	9 (56%)	0.787
Male	40 (49%)	7 (44%)	
BMI (kg/m^2^)	32 (19–53)	28 (18–50)	0.121
Diabetes			
No	57 (70%)	8 (50%)	0.147
Yes	24 (30%)	8 (50%)	
CAD			
No	73 (91%)	15 (94%)	1.000
Yes	7 (9%)	1 (6%)	
Previous hernia repair			
No	47 (59%)	6 (40%)	0.258
Yes	33 (41%)	9 (60%)	
Previous mesh implantation			
No	46 (61%)	6 (46%)	0.366
Yes	29 (39%)	7 (54%)	
Smoking status			
Never	38 (47%)	7 (44%)	1.000
Current or former	43 (53%)	9 (56%)	
Recent smoking status			
Never smoked	64 (79%)	13 (81%)	1.000
Current smoker or quit during the past year	17 (21%)	3 (19%)	
Etiology of hernia			
Incisional	81 (100%)	16 (100%)	
Location of hernia			
ventral	79 (98%)	16 (100%)	1.000
f lank	2 (2%)	0 (0%)	
Hernia Volume (cm^3^)	**2316 (140–24314)**	**4221 (1398–24232)**	**0.077**
Abdominal Cavity Volume (cm^3^)	46918 (18982–113636)	50580 (19698–112922)	0.697
Hernia-to-abdominal Cavity Ratio (%)	5 (0–48)	7 (3–99)	0.108
Pre-operative Botox injection			
No	**47 (58%)**	**13 (81%)**	**0.097**
Yes	**34 (42%)**	**3 (19%)**	
Complication associated with Botox injection			
No	35 (100%)	3 (100%)	
Botox-to-surgery time (days)	30 (8–58)	31 (21–38)	0.933
Retrorectus surgical technique			
No	16 (20%)	6 (38%)	0.187
Yes	65 (80%)	10 (62%)	
Primary fascial closure			
No	6 (7%)	1 (6%)	1.000
Yes	75 (93%)	15 (94%)	
Delayed closure			
No	75 (93%)	14 (88%)	0.615
Yes	6 (7%)	2 (12%)	
Surgical component separation			
No	55 (68%)	11 (69%)	1.000
Yes	26 (32%)	5 (31%)	
Application of mesh			
No	7 (9%)	1 (6%)	1.000
Yes	74 (91%)	15 (94%)	
Mesh type			
None	**7 (9%)**	**1 (6%)**	**0.020**
Polypropylene (Prolene)	**41 (51%)**	**4 (25%)**	
Synthetic Biodegradable (Vicryl, Phasix)	**8 (10%)**	**4 (25%)**	
Composite Mesh (Bard Ventralex)	**19 (23%)**	**2 (12%)**	
Biologic (Strattice)	**6 (7%)**	**5 (31%)**	
Additional intraoperative procedures other than hernia repair			
No	62 (77%)	12 (75%)	1.000
Yes	19 (23%)	4 (25%)	
Need for blood transfusion during surgery			
No	79 (99%)	15 (94%)	0.307
Yes	1 (1%)	1 (6%)	
Estimated Blood Loss (ml)	100 (10–1000)	75 (15–1000)	0.807
Hospital stay (days)	**3 (0–20)**	**7 (2–14)**	**0.002**
Discharge destination			
Home	**68 (84%)**	**10 (62%)**	**0.088**
Home w/Assistance	**8 (10%)**	**5 (31%)**	
Subacute Rehab	**3 (4%)**	**1 (6%)**	
Inpatient rehab	**2 (2%)**	**0 (0%)**	
Any complications			
No	**64 (81%)**	**1 (6%)**	** < 0.001**
Yes	**15 (19%)**	**15 (94%)**	
Post-operative pain control issues			
No	**80 (99%)**	**13 (81%)**	**0.014**
Yes	**1 (1%)**	**3 (19%)**	
Reoperation for hernia repair during hospitalization			
No	81 (100%)	16 (100%)	na
30-day hernia reoperation			
No	81 (100%)	16 (100%)	na
Alive at last follow-up?			
Yes	81 (100%)	16 (100%)	na
Follow-up duration (months)	43 (2–81)	48 (4–79)	0.228

1. Data summarized as frequency (%) for categorical variables and median (min – max) for numerical values

2. Percentages are compared using the Fisher's exact test, and the median values are compared with the Mann–Whitney U test

3. Statistically significant differences are shown in bold. Statistical significance level is set at 0.10

In multivariate logistic regression analysis chemical component separation with Botox was associated with significantly decreased risk of hernia recurrence (OR [95% confidence interval]: 0.06 [0.01–0.67], *p* = 0.021) (Table 3).

**Table 3:** Factors associated with hernia recurrence in a multivariate logistic regression model. Variables selection has been conducted using the forward stepwise method

	Forward stepwise selection^1^		Final logistic model^2^	
	OR (95% CI)	*p* value	OR (95% CI)	*p* value
Chemical component separation with Botox	**0.08 (0.01–0.59)**	**0.013**	**0.06 (0.01–0.67)**	**0.021**
Hernia volume (cm^3^)^3^	**2.94 (1.00–8.62)**	**0.050**	**6.70 (0.92–48.87)**	**0.061**
Mesh type (composite versus biologic)	**0.12 (0.01–1.07)**	**0.057**	0.09 (0.00–2.03)	0.132
Any postoperative surgical complications	**248.44 (13.45–4588.14)**	** < 0.001**	**32.89 (0.96–1125.00)**	**0.053**

1. Included variables were chemical component separation with Botox, body mass index, postoperative length of hospital stay, hernia volume, hernia-to-abdominal cavity volume ratio, type of implanted mesh, postoperative discharge destination, any postoperative surgical complications, postoperative pain control issues and postoperative follow-up duration

2. Multivariate logistic regression including the natural logarithm of follow-up duration as the offset variable and adjusted for a significant interaction detected between chemical component separation and mesh type

3. Standardized value is included in the analysis

### Propensity score-weighted analysis

After propensity score weighting, chemical component separation with Botox injection demonstrated approximately 71% ± 21% reduced risk of hernia recurrence relative to the non-Botox group (adjusted base recurrence risk in non-Botox group ± standard error: 18% ± 5%, absolute risk reduction in the Botox group: −13% ± 6%; *p* = 0.045) (Fig. 4).

## Discussion

Using a propensity score-weighted analysis, this study suggests that chemical component separation with bilateral CT-guided administration of Botox into the muscle layers involved in the ventral hernia may decrease postoperative hernia recurrence rates.

The effectiveness of preoperative Botox injection in reducing hernia defect size and increasing primary closure rates has been demonstrated in a few systematic review and meta-analysis studies [17–20]. The preoperative measurements of the hernia defect have been shown to correlate with the patient’s risk of recurrence after surgical repair [21]. Studies with variable follow-up durations (1–49 months) have reported 0–11.4% recurrence rates with preoperative chemical component separation (CCS) with Botox injections [17]. This is comparable to the recurrence rate of 8% demonstrated in our study.

Only a small number of comparative studies have assessed effectiveness of preoperative Botox injection on operative success indices of hernia repair in comparison to adjunctive surgical techniques. In a prospective study comparing 40 patients with Botox injection and subsequent open Rives repair with another 40 patients with open component separation, Botox injection was associated with lower rates of surgical site complications. In the same study, 2 recurrences occurred during a median of 19.6 months, none of which was in the Botox group [22]. Three propensity score-matched retrospective studies demonstrated comparable recurrence rates between Botox and non-Botox groups [14, 23, 24]. In one retrospective cohort of 426 patients, Botox injection was associated with comparable rates of fascial closure when compared to non-Botox group despite significantly larger fascial defect sizes in the Botox group. On the other hand, higher rates of surgical site infection/occurrences were observed in the Botox group [25]. CCS with Botox injection was associated with lower rates of surgical site occurrences in multivariate analysis in a propensity score-matched study [24]. No significant differences in overall complication rates were observed in our study.

CCS exerts its effect through an anticholinergic mechanism, achieving peak flaccid paralysis as soon as 2 weeks and lasting up to 6 weeks [10]. This adjunct results in a smaller preoperative hernia defect which minimizes the intraoperative tension required at the repair site and avoids the'cheese-wiring'effect caused by excessive tension that can lead to dehiscence [7, 26]. In a prospective study, maximum hernia defect size reduction was achieved by 3 weeks after Botox injection and no recurrence was observed during 9 months after repair [27]. This effect has been shown to last 2–3 months and gradually fade away during the subsequent 6–9 months [28].

A consensus proposal has recently been made by the Spanish Association of Surgeons regarding use of Botox injection prior to abdominal wall surgery and proposing a standard informed consent form for the procedure [29].

The possible effect of surgical technique on rates of recurrence is an important consideration. One Randomized Controlled study of 56 patients comparing transversus abdominis muscle release versus mesh-only repair demonstrated no statistically significant difference in recurrence rates [30]. However, in one retrospective study using the Abdominal Core Health Quality Collaborative registry, surgical component separation was associated with lower overall recurrence rates [31]. It is confirmed that achieving primary closure significantly reduces recurrence rates, but it remains unconfirmed whether surgical component separation alone has the same association. Since there was no pre-planned classification of surgical techniques, a higher rate of surgical component separation in the Botox group may be explained by several factors. One possibility is that larger hernias were preferentially assigned to the Botox group which is possibly the explanation for a similar difference seen in a previous study [25]. Though our data did not indicate a significant size discrepancy. Another explanation is that the hernias in the Botox group may have had other unmeasured characteristics that influenced surgical decision-making. Additionally, observer bias or the Hawthorne effect [32] may have played a role, with surgeons potentially opting for component separation more frequently when aware that the patient had received a Botox injection. In our study, evaluation of association between primary fascial closure and recurrence rates was not possible due to an overall high rate of primary fascial closure. An important consideration in our study is that propensity score weighting was applied to minimize potential confounding effects of surgical component separation and follow-up duration.

There is no significant difference in primary fascial closure rates between two groups in the current study. This might be a signal that the smaller number of hernia recurrences in the CCS group may be independent of primary closure.

This study has a number of strengths. First, potential effect of shorter follow-up duration has been minimized via fixation of time effect in the statistical models via offsetting in the regression models and including it in the propensity score calculation. This difference has been observed in previous studies with potential to underestimate recurrence rates in the Botox group. Additionally, attempts have been made to minimize potential confounding effects of significant technical differences at baseline between two study arms using statistical techniques. To this end, doubly-robust propensity score weighting technique has been chosen to achieve the optimum weighting with the minimum statistical estimation bias.

On the other hand, this study is inherently limited by its retrospective design and single-institution setting, leaving it vulnerable to unobserved selection bias. Also, relatively small sample size leaves the study with low statistical power especially in the initial comparative analyses. Additionally, although we have tried to minimize selection bias, multiple confounding factors such as variability in surgical techniques and perioperative variables could not be addressed due to the retrospective nature of the study.

In conclusion, this study suggests that chemical component separation with OnabotulinumtoxinA (Botox) can be an effective method in reducing recurrence rates in large complex ventral hernias. Further prospective studies can be considered to validate this finding.

## Data Availability

De-identified data and analysis code are available from the corresponding author upon request and pending institutional approval.
